# Diagnostic and Prognostic Value of *SHOX2* and *SEPT9* DNA Methylation and Cytology in Benign, Paramalignant and Malignant Pleural Effusions

**DOI:** 10.1371/journal.pone.0084225

**Published:** 2013-12-27

**Authors:** Dimo Dietrich, Maria Jung, Svenja Puetzer, Annette Leisse, Emily Eva Holmes, Sebastian Meller, Barbara Uhl, Philipp Schatz, Claudia Ivascu, Glen Kristiansen

**Affiliations:** 1 University Hospital Bonn (UKB), Institute of Pathology, Bonn, Germany; 2 Metanomics Health GmbH, Berlin, Germany; 3 Roche Pharma AG, Hematology/Oncology, Grenzach-Wyhlen, Germany; Kinghorn Cancer Centre, Garvan Institute of Medical Research, Australia

## Abstract

Pleural effusions (PE) are a common clinical problem. The discrimination between benign (BPE), malignant (MPE) and paramalignant (PPE) pleural effusions is highly important to ensure appropriate patient treatment. Today, cytology is the gold standard for diagnosing malignant pleural effusions. However, its sensitivity is limited due to the sometimes low abundance of tumor cells and the challenging assessment of cell morphology in cytological samples. This study aimed to develop and validate a diagnostic test, which allows for the highly specific detection of malignant cells in pleural effusions based on the DNA methylation biomarkers *SHOX2* and *SEPT9*. A quantitative real-time PCR assay was developed which enabled the accurate and sensitive detection of *SHOX2* and *SEPT9* in PEs. Cytological and DNA methylation analyses were conducted in a case control study comprised of PEs from 114 patients (58 cases, 56 controls). Cytological analysis as well as *SHOX2* and *SEPT9* methylation resulted in 100% specificity. 21% of the cases were cytologically positive and 26% were *SHOX2* or *SEPT9* methylation positive. The combined analysis of cytology and DNA methylation resulted in an increase of 71% positively classified PEs from cancer patients as compared to cytological analysis alone. The absolute sensitivity of cytology and DNA methylation was not determinable due to the lack of an appropriate gold standard diagnostic for distinguishing between MPEs and PPEs. Therefore, it was unclear which PEs from cancer patients were malignant (containing tumor cells) and which PEs were paramalignant and resulted from benign conditions in cancer patients, respectively. Furthermore, DNA methylation analysis in PEs allowed the prognosis of the overall survival in cancer patients (Kaplan-Meier analysis, log rank test, p = 0.02 (*SHOX2*), p = 0.02 (*SEPT9*)). The developed test may be used as a diagnostic and prognostic adjunct to existing clinical and cytopathological investigations in patients with PEs of unclear etiology.

## Introduction

Pleural effusions (PEs) represent a common clinical complication. Most commonly, PEs are caused by cardiac failure, pneumonia, pulmonary embolism and malignant neoplasm [1], [2]. Clinical problems, which result from PEs, are dyspnea, pleural space infections, pleural empyema, and pleural scar tissues [3], [4], [5]. A therapeutic release of the PE is indicated.

Malignancies are the main cause for PEs [2], [6]. 50% up to 65% of malignancies causing PEs are lung and breast cancers [7]. Malignant pleural effusions (MPEs) may be the initial manifestation of a cancer or may develop during the advanced phases of a known malignancy. The presence of MPEs in cancer patients is associated with an overall poor prognosis leading to an overall survival of less than one year [8], [9], [10]. Treatment of these patients is always palliative [11], [5]. However, not all cancer patients with a PE appear to have a MPE, but show paramalignant pleural effusions (PPEs). These MPEs occur since cancer patients frequently develop comorbidities such as heart failure, pneumonia, or pulmonary embolism, each of them possibly causing an effusion [11]. Reliable figures regarding the relative number of PPEs and MPEs cannot be found in the literature due to the lack of an accurate gold standard method to discriminate between PPEs and MPEs. A careful evaluation of the effusion is therefore necessary to establish its etiology and to direct the patients’ therapy. The discrimination between MPE and PPE might direct a decision towards curative or palliative treatment [11] and is considered for accurate tumor staging according to the TNM-classification.

The gold standard for diagnosing MPEs is cytology. While it is a highly specific diagnostic tool with up to 100% specificity [12], [13], its sensitivity is limited. The diagnostic sensitivity of cytological analysis averaged over all tumor entities ranges from 40% up to 90% and highly depends on the origin of the primary tumor [2], [11], [14], [15]. Only few studies analyze cytology with regard to specific tumor entities and report sensitivities ranging from 18% for lymphoma to 83% for ovarian cancer [16].

Furthermore, the sensitivity of cytological studies depends on the volume of the pleural fluid sample, the number of specimens, the type of preparation and the experience of the examiner [12], [2]. Nevertheless, it is difficult to discriminate malignant from benign cells by morphology in the pleural fluid due to mesothelial and macrophage abnormalities. Actively dividing mesothelial cells for example can mimic adenocarcinoma.

New diagnostic approaches to increase the sensitivity for MPEs are urgently needed. Molecular biomarkers have the potential for improving the clinical management of cancer patients and patients who are suspected of having cancer. A few studies on biomarkers for the discrimination between MPE and BPE have been published so far. The clinical performance of mesothelin, SMRP, survivin, CEA, CA 15-3, CA 19-9, CYFRA 21-1, NSE, TSA have been tested [17], [18], [19] but the sensitivity did not exceed 35% at 100% specificity [19].

DNA methylation is a valuable source for cancer biomarkers. Methylation of the cytosine within the CpG dinucleotide context plays a key role in fundamental biological processes and human diseases and aberrant DNA methylation is a hallmark of human cancers [20], [21], [22], [23]. DNA itself is a chemically robust molecule and DNA methylation is a stable mark. Therefore, DNA methylation biomarkers represent an ideal target for use in clinical routine. However, until now, only one study reported the utility of methylation biomarkers for the diagnosis of MPEs [13].

*SHOX2* and *SEPT9* are among the most validated DNA methylation markers reported so far. Both biomarkers are already in use in diagnostic tests for lung and colorectal cancer, respectively. Aberrant DNA methylation of *SHOX2* is a hallmark of lung cancer tumors and correlates to an amplification of the respective locus 3q25.3 [24], [25]. Methylation of the *SHOX2* gene locus in bronchial fluid aspirated during bronchoscopy is a validated biomarker in patients with suspected lung cancer and allowed for accurate detection of malignant lung disease even in patients with a negative cytopathological result and no visible tumor in bronchoscopy [26], [27]. Furthermore, *SHOX2* DNA methylation in plasma is a sensitive and specific biomarker for detecting lung cancer. Sensitivity was particularly high for small cell lung cancer and squamous cell carcinoma [28]. A CE-marked *in vitro* diagnostic (IVD) test to aid pathologists in the diagnosis of lung cancer based on the *SHOX2* DNA methylation biomarker is commercially available in Europe [27].

*SEPT9* DNA methylation has been reported to be a powerful biomarker for colorectal cancer [29], [30], [31]. *SEPT9* DNA methylation occurs early on during carcinogenesis and can already be found in precancerous lesions, i.e. adenomas [32]. Thus, the analysis of *SEPT9* DNA methylation in blood plasma is a promising test for colorectal cancer screening [29], [30], [31]. The *SEPT9* DNA biomarker was recently validated in a large observational prospective colorectal cancer screening trial (PRESEPT, ClinicalTrials.gov Identifier: NCT00855348, http://clinicaltrials.gov/ct2/show/NCT00855348) involving nearly 8,000 asymptomatic subjects scheduled to have a colonoscopy [33].

It is well acknowledged that DNA methylation biomarkers are usually not specifically methylated in only a certain tumor entity [34]. *SHOX2* for example is methylated in the different histological subtypes of lung cancer [24], [26], [27], [28]. *SEPT9* is methylated in colorectal adenocarcinoma and frequently in head and neck squamous cell carcinomas [35]. Hence, it is likely that *SHOX2* and *SEPT9* are methylated in several different malignancies and represent promising pan cancer biomarkers in clinical questions where the discrimination between malignant and benign disease irrespective of any specificity regarding the origin of a malignant tumor is desired.

The goal of this study was to test the potential of the *SHOX2* and *SEPT9* DNA methylation biomarkers to improve the sensitivity for detecting malignant cells in PEs and to allow for an accurate prognosis in these patients. In combination with cytology such an assay might significantly improve the clinical management of patients with PE and may be used as a diagnostic adjunct to existing clinical and cytopathological investigations.

## Materials and Methods

### Ethics Statement

The study has been approved by the Institutional Review Board (IRB) at the University Hospital of Bonn. Informed consent (written) was obtained from all donors or their next of kin.

### Patients

Both cancer patients and patients of the control group donating samples for this study were investigated for suspected cancer at the same clinics at the University Hospital of Bonn between 09/2012 and 07/2013. All PE samples were collected under ultrasound guidance to locate the pleural effusion with a 30 G needle [Becton Dickinson and Company, NJ, USA] under aspiration. PE specimens were fixed with equal volume of Saccomanno’s fixative and stored at room temperature. The characteristics of the patients included in this study are shown in **Table 1** and **Table 2**. The presence of malignant disease was confirmed by histology based on biopsy or surgical specimen or by cytology. In one case medical imaging showed a malignant tumor which was not confirmed by histology due to the patient’s health condition. Benign conditions were tested by means of microbiological diagnostics, ultrasound, cardiac catheterisation, and x-ray. Only patients who did not have any cancer related history within the last 15 years were considered for the control group. For cytopathological assessment, cytospins or smear preparations from PEs were prepared. The cellular fraction from PE samples containing a high number of cells were formalin-fixed and paraffin-embedded. Sections, cytospins, and smear preparations were analyzed using one or more of the following staining protocols: hematoxylin and eosin (HE), thrombomodulin, TTF1, EpCAM, Periodic Acid Schiff (PAS), Papanicolaou's (PAP), and/or May-Gruenwald-Giemsa (MGG).

**Table 1:** Characteristics of the patient population.

	Total	Cancer Cases	Controls
**Age**	114 (100%)	58 (100%)	56 (100%)
≤ 50 Years	14 (12%)	4 (7%)	10 (18%)
51–60 Years	11 (10%)	7 (12%)	4 (7%)
> 60 Years	89 (78%)	47 (81%)	42 (75%)
Median Age [Years]	71.5	70	73.5
Age Range [Years]	23–92	30–87	23–92
**Follow-up**			
Death	X	22 (38%)	X
Alive	X	36 (62%)	X
Mean Follow-up [Days]	X	62	X
Median Follow-up [Days]	X	36	X
Range [Days]	X	0–250	X
**Gender**			
Female	44 (39%)	25 (43%)	19 (34%)
Male	70 (61%)	33 (57%)	37 (66%)
**Non-Malignant Disease**			
Heart Diseases	45 (40%)	22 (38%)	23 (41%)
Cardiac Decompensation and Heart Failure	21 (18%)	5 (9%)	16 (29%)
Pneumonia	18 (16%)	6 (10%)	12 (21%)
Renal Failure	18 (16%)	6 (10.%)	12 (21%)
Sepsis	15 (13%)	6 (10%)	9 (16%)
Lung Diseases	9 (8%)	4 (7%)	5 (9%)
Gastrointestinal Diseases	8 (7%)	2 (3%)	6 (11%)
Hepatic Failure	6 (5%)	1 (2%)	5 (9%)
Stroke	6 (5%)	5 (9%)	1 (2%)
Infectious Diseases	4 (4%)	1 (2%)	3 (5%)
Pancytopenia, Anemia	2 (2%)	0 (0%)	2 (4%)
Others (BPH, Hypothyroidism, etc.)	9 (8%)	5 (9%)	4 (7%)
**Cytology Result**			
Positive	12 (11%)	12 (21%)	0 (0%)
Negative	92 (81%)	39 (67%)	53 (95%)
Suspicious	10 (9%)	7 (12%)	3 (6%)

Clinical data of the 114 patients (58 cancer cases, 56 controls) included into the case control study.

**Table 2:** Site (organ) specificity of malignant diseases in 58 cancer cases.

Organ	No of Patients (%)
All Sites	58 (100%)
Digestive System	17 (29%)
Stomach	3 (5%)
Small Intestine	1 (2%)
Colon	4 (7%)
Anus, Anal Canal, & Anorectum	1 (2%)
Liver & Intrahepatic Bile Duct	7* (12%)
Pancreas	1 (2%)
Respiratory System	10 (17%)
Larynx	2 (3%)
Lung & Bronchus	8* (14%)
Bones & Joints	1* (2%)
Skin (Excluding Basal & Squamous)	1 (2%)
Melanoma-skin	1 (2%)
Breast	11* (19%)
Genital System	7 (12%)
Uterine Cervix	1* (2%)
Ovary	5 (9%)
Prostate	1 (2%)
Urinary System	5 (9%)
Kidney & Renal Pelvis	4 (7%)
Ureter & other Urinary Organs	1 (2%)
Brain & other Nervous System	1 (2%)
Endocrine System	2 (3%)
Thyroid	2 (3%)
Lymphoma	5 (9%)
Non-Hodgkin Lymphoma	3* (5%)
Myeloma	2 (3%)
Leukemia	2 (3%)
Acute Myeloid Leukemia	1* (2%)
Chronic Myeloid Leukemia	1* (2%)
Other & Unspecified Primary Sites	1 (2%)

one patient with lung, uterine cervix cancer and non-Hodgkin lymphoma, one patient suffering from lung and liver cancer, one patient with breast and bone cancer, and one patient with acute and chronic myeloid leukemia and breast cancer.

### Sample and Calibrator Preparation

A calibrator sample (bisulfite-converted artificially methylated human DNA) was prepared as follows: 80 µl methylated DNA (100 ng/µl, CpGenome™ Universal Methylated DNA, Millipore, Billerica, MA, USA), 80 µl bisulfite reagent (65 % ammonium bisulfite, pH 5.3 [TIB Chemicals, Mannheim, Germany]) and 40 µl denaturation reagent (0.1 g/ml hydrochinone [Sigma-Aldrich, St. Louis, MO, USA] in DEG [diethylene glycol, Sigma-Aldrich, St. Louis, MO, USA]) were mixed and the mixture was incubated for 45 min at 85°C and 1,000 rpm in a thermomixer. The converted DNA was purified by means of Nanosep centrifugal devices (10K, PALL, East Hills, NY, USA) as previously described [36]. The DNA concentration of sperm and universal methylated DNA was determined by UV spectrophotometry using a Nanodrop ND-1000 spectral photometer (Nanodrop Technologies, Wilmington, DE, USA).

For cut-off validation and analytical performance evaluation, DNA samples with defined methylation levels were prepared by mixing DNA from washed human research sperm (NW Andrology & Cryobank Inc., Spokane, WA, USA) and CpGenome™ Universal Methylated DNA. DNA from sperm was extracted using the QIAamp DNA Micro Kit (Qiagen, Hilden, Germany) following the manufacturer’s recommendations. The DNA mixtures were bisulfite converted as described above.

The cellular fractions of the PEs were pelleted by centrifugation for 10 min at 4000 x g. The cellular pellet was washed twice with PBS buffer. For sample lysis 100 µl lysis buffer (50 mM Tris-HCl, pH 8.4, 1 mM EDTA, 0.5 % [v/v] TWEEN®20) and 10 µl 2 % proteinase K in lysis buffer were added to each cellular pellet. The mixture was incubated overnight at 60°C and 1,000 rpm in a thermomixer. 10 µl proteinase K (2 % [w/v]) in lysis buffer were added and the mixture was incubated for additional 4 h at 60°C and 1,000 rpm in a thermomixer. 100 µl of this mixture were then mixed with 100 µl bisulfite reagent and 50 µl denaturation buffer and incubated for 45 min at 85°C and 1,000 rpm in a thermomixer. 500 µl binding buffer (50 % ethanol, 50 % guanidiniumthiocyanate solution (6 M in 0.1 M Tris; pH 7.5) [AppliChem GmbH, Darmstadt, Germany]) were added to the reaction mixture. The mixture was transferred onto a NucleoSpin Extract II Column (Macherey & Nagel, Dueren, Germany) and centrifuged at 14,000 x g for 3 min. The bound DNA was washed with 700 µl wash buffer (10 % Trizma® HCl [Sigma-Aldrich, St. Louis, MO, USA], 90 % ethanol) followed by centrifugation (14,000 x g, 1 min). The DNA was desulphonated with 700 µl alkaline buffer (250 mM NaOH [AppliChem GmbH], 75 % ethanol). The column was centrifuged at 14,000 x g for 1 min. After this desulphonation, the membrane was washed twice with wash buffer (1^st^: 700 µl wash buffer, 1 min at 14,000 x g; 2^nd^: 500 µl wash buffer, 3 min at 14,000 x g). The silica membrane was dried 10 min at 60°C with open lids. Bisulfite converted DNA was eluted with 70 µl elution buffer (1 % Tris-HCl 1M pH 8.0 [AppliChem GmbH]) for 1 min at 14,000 x g.

### Real-time PCR Quantification of *SHOX2* and *SEPT9* DNA Methylation

PCR reaction were carried out in 10 µl volumes with the following composition: 70 mM Tris-HCl, pH 8.4, 12 mM MgCl_2_, 100 mM KCl, 8 % glycerol, 0.5 mM each dNTP, 2 U FastStart Taq DNA polymerase [Roche Applied Science, Penzberg, Germany], 0.006 µl ROX solution. The ROX solution was prepared as previously described [24]. Oligonucleotides (**Table S1**) for the triplex assay were modified and optimized based on previously published *SEPT9*, *SHOX2* and *ACTB* assays [24], [31], [37]. 25 ng template DNA according to UV quantification was used as template DNA per each single PCR reaction.

PCR was performed using an AB 7500 Fast Real-Time PCR System (Life Technologies Corporation, Carlsbad, CA, USA) using the following temperature profile: 20 min at 95°C followed by 50 cycles with 2 s at 62°C, 45 s at 56°C (each at 100 % ramp rate) and 15 s, 95°C (at 75 % ramp rate). Thresholds and baselines were set as follows: 0.015 (threshold *SHOX2*), 0.01 (threshold *SEPT9*), 0.02 (threshold *ACTB*), 3–24 (baseline).

The specificity of the assay for bisulfite converted DNA was confirmed using non-converted genomic template DNA.

### Data Evaluation and Statistical Analysis

Each sample and the calibrator was analysed in triplicate. Sample determinations were considered to be valid when the median of the CT-values met the following quality criterion: 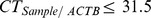 [23], 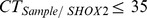, or 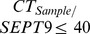. Other samples were excluded from analysis due to insufficient DNA yield.

For each sample, a relative methylation value was determined using the ΔΔCT method adapted for DNA methylation analyses as previously described [24]. In brief, ΔΔCT values (here exemplarily shown for *SHOX2*) were computed as follows: 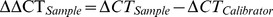, where 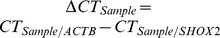 and 
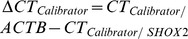
. ΔΔCT values for *SEPT9* were computed accordingly. Percentage methylation was calculated using the following formula: 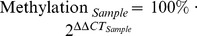 [24].

For *SHOX2*, a methylation cut-off was assigned for dichotomization of the methylation value. Samples showing a relative *SHOX2* methylation level above the cut-off were classified as *SHOX2* positive, all others were classified as negative.

Students t-Tests were performed in order to compare *SHOX2* and *SEPT9* methylation levels of samples of control and cancer patients. Kaplan-Meier analysis was conducted to investigate the overall survival (OS). Overall survival time indicates the time difference between the date of the release of the pleural effusion and the date of death or the last contact, respectively. OS were compared using the log rank test. A correlation between *SHOX2* and *SEPT9* methylation was tested using a Pearson’s correlation. Analysis of variance (ANOVA) was conducted to check for an inter-run variability of test results. All statistical analyses were performed using the SPSS software version 21 (IBM, Armonk, NY, USA). P-values <0.05 were considered statistically significant.

## Results

### Assay Principle and Analytical Performance Evaluation

A quantitative real-time PCR triplex assay was developed to accurately and simultaneously assess the methylation level of *SHOX2* and *SEPT9* DNA in PEs. Methylation-specific PCR (MSP) [38] together with the TaqMan™ probe technology for real-time PCR detection were used. Quantification of total DNA content was based on the β-actin gene (*ACTB*) in a methylation independent manner. All assays (*SHOX2*, *SEPT9* and *ACTB*) were performed in a single-tube triplex real-time PCR assay.

In order to investigate the analytical performance of the assay unmethylated DNA was spiked with different amounts of artificially methylated DNA. Both, methylation levels of *SEPT9* and *SHOX2*, were assessed with high precision and accuracy (**Figure S1**). The assay showed a dynamic range between 0% and 100% methylation and was therefore suitable for quantifying methylation levels of *SHOX2* and *SEPT9*.

### Clinical Performance

The developed assay was used in a case control study comprised of PEs from 114 patients (58 cancer patients, 56 control patients with benign diseases). Patient samples yielding insufficient DNA for an accurate methylation analysis were excluded. Therefore, the previously described *ACTB*-CT criterion [27] was combined with predefined exclusion criteria based on *SHOX2* and *SEPT9* CT values. Since malignant cells in PEs usually represent a minor fraction of the total cells, a low input amount decreases the probability of the presence of DNA from malignant cells for statistical reasons and therefore leading to a false negative result. Out of the 114 analyzed patient samples, 80 yielded sufficient DNA according to the previously described sample exclusion criteria.

**Figure 1:**
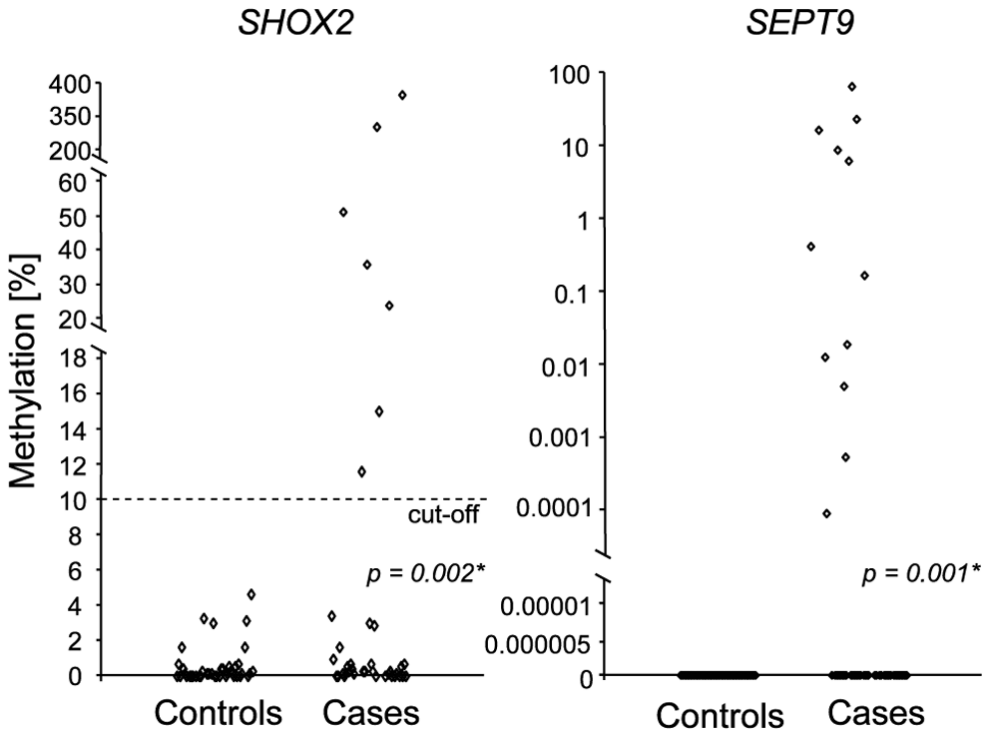
*SHOX2* and *SEPT9* DNA methylation in PEs from cancer patients and patients with benign diseases. Methylation values of *SHOX2* and *SEPT9* in PEs from patients with cancer (cases) and patients with benign diseases (controls) determined by quantitative real-time PCR. The p-values refer to a Students t-test. A methylation cut-off was used to classify patient samples as *SHOX2* positive (above the cut-off).

**Figure 2:**
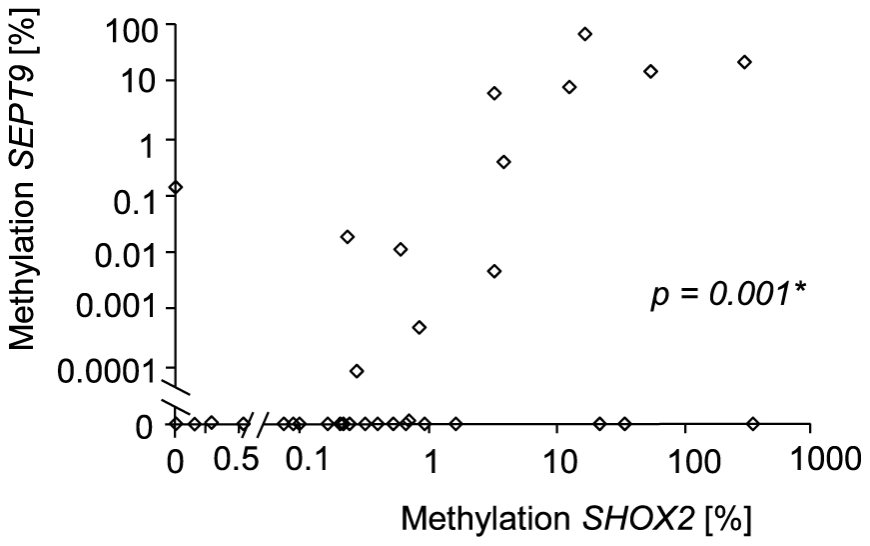
Correlation of *SHOX2* and *SEPT9* DNA methylation in PEs. Correlation of the methylation values of *SHOX2* and *SEPT9* in PEs from cancer patients. The p-value refers to a Pearson’s correlation.

As shown in **Figure 1** **,** low level methylation of *SHOX2* was also found in samples from patients with benign diseases. Therefore, the introduction of a methylation cut-off was required in order to dichotomize the results and to transfer the quantitative result into a qualitative result. Out of 58 cancer patient samples, 12 (21%) showed detectable *SEPT9* methylation and seven (12%) showed *SHOX2* DNA methylation levels above the cut-off and were defined as methylation positive. The methylation levels of *SHOX2* and *SEPT9* correlated significantly with each other (**Figure 2**). 15 out of 58 cancer patient samples (26%) showed *SHOX2* or *SEPT9* methylation positivity (**Figure 1** **,** **Table 3**). Positive results in 21 out of 58 cases (36%) were obtained by the combination of cytological and DNA methylation analyses. Therefore, the number of positively classified PEs from cancer patients increased by 71% when combining DNA methylation analysis and cytology as compared to cytological assessment alone. All control patients were methylation negative and showed a negative cytology leading to a specificity of 100%.

**Table 3:** Clinical performance of the DNA methylation biomarkers *SHOX2* and *SEPT9* and cytology in PEs from 114 patients.

Diagnostic Result	Total Number	Cases	Controls	Positivity	Specificity
**Cytology +**	12/114	12/58	0/56	21%	
**Cytology** –	92/114	39/58	53/56		100%
**Cytology (+)**	10/114	7/58	3/56		
***SHOX2*** ** +**	7/114	7/58	0/56	12%	
***SHOX2*** –	73/114	31/58	42/56		100%
***SHOX2*** ** nd**	34/114	20/58	14/56		
***SEPT9*** ** +**	12/114	12/58	0/56	21%	
***SEPT9*** –	68/114	26/58	42/56		100%
***SEPT9*** ** nd**	34/114	20/58	14/56		
***SEPT9*** ** or ** ***SHOX2*** ** +**	15/114	15/58	0/56	26%	
***SEPT9*** ** and ** ***SHOX2*** –**/nd**	99/114	43/58	56/56		100%
***SEPT9*** ** or ** ***SHOX2*** ** or Cytology +**	21/114	21/58	0/56	36%	
***SEPT9*** ** and ** ***SHOX2*** ** and Cytology** –**/(+)/nd**	93/114	37/58	56/56		100%

Positive test results are labelled “+”, cytologically suspicious for malignancy were labelled by “(+)”. Negative test results are labelled by “–”. Invalid test results due to low DNA content are shown as “nd”. The results classified as “(+)” and “nd” were used equivalently to negative test results in the calculation of positivity and specificity.

Hypermethylation was detected in PEs of patients with different cancer types (**Table S2**). The highest *SHOX2* methylation levels were found in samples from patient with breast (*SHOX2*: 363%, *SEPT9*: 0%), a patient with stomach (*SHOX2*: 270%, *SEPT9*: 22%) and a patient with lung cancer (*SHOX2*: 51%, *SEPT9*: 16%). Highest *SEPT9* levels were found in PE samples from two patients with stomach cancer (*SHOX2*: 16%, 270%, *SEPT9*: 65%, 22%) and a patient suffering from lung cancer (*SHOX2*: 51%, *SEPT9*: 16%).

**Figure 3:**
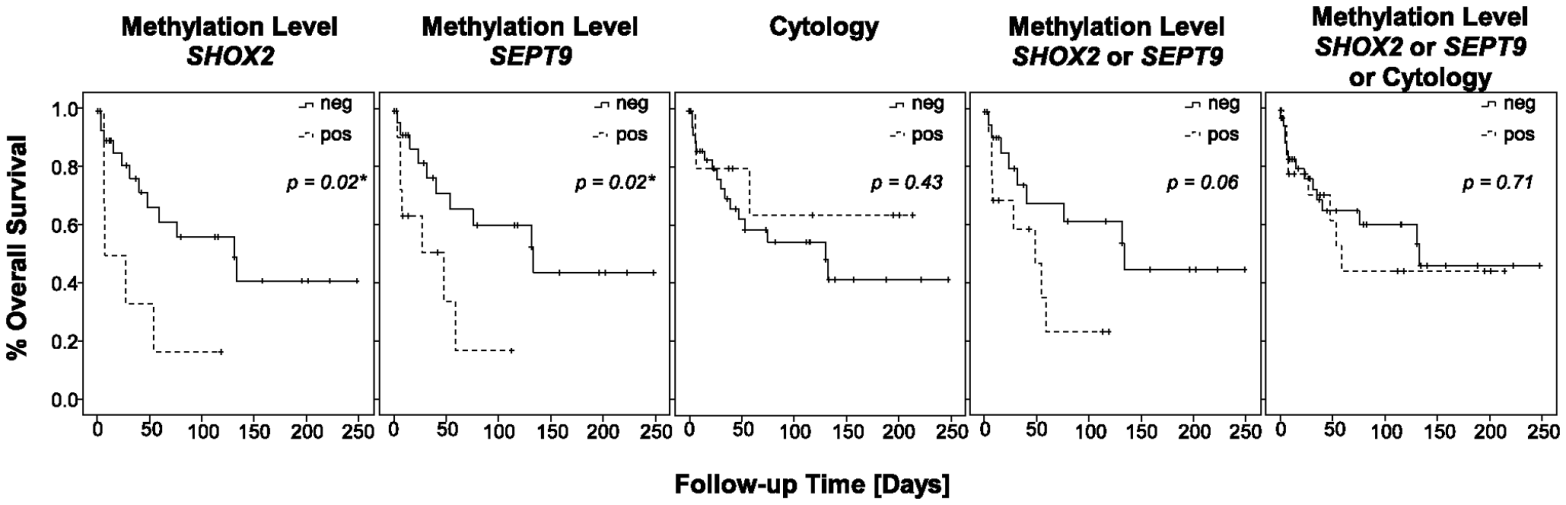
Survival analyses. Kaplan-Meier analysis of overall survival in 58 cancer patients stratified by the cytological diagnosis and the DNA methylation status of *SHOX2* and *SEPT9* in PEs. The p-values refer to the log rank test.

Patients with a MPE are expected to have a poorer outcome as compared to patients with PPE since patients with MPE usually present at more advanced stages. Therefore, a Kaplan-Meier survival analysis of the cancer patients stratified by the *SHOX2* and *SEPT9* methylation levels was conducted. **Figure 3** shows that cancer patients with a *SHOX2* or *SEPT9* methylation positive PE have a significantly shorter overall survival as compared to cancer patients with a methylation negative PE. A positive cytological result was not a significant predictor for adverse outcome. This finding supports the hypothesis that methylation positivity in PEs from cancer patients allows for the discrimination between malignant and paramalignant PEs.

### Cut-off Validation

**Figure 4:**
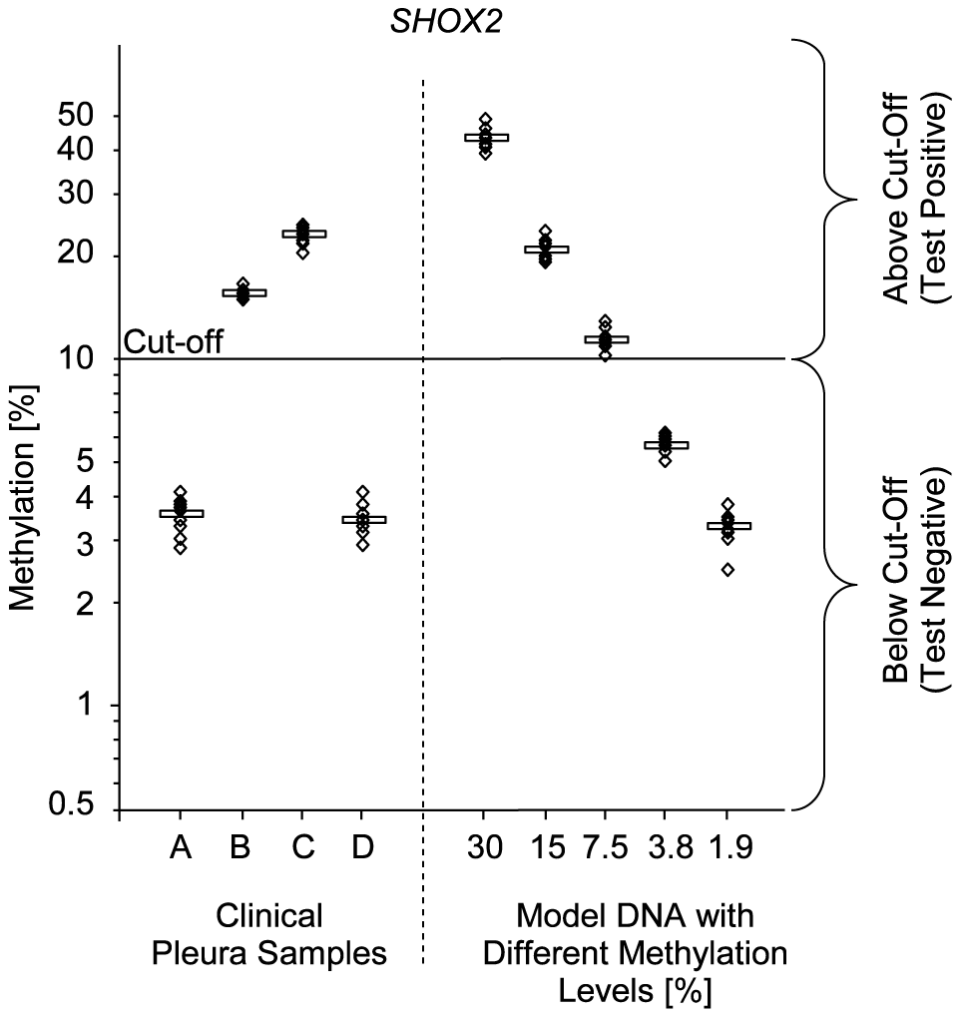
Cut-off validation. Cut-off validation for the *SHOX2* diagnostic assay. Model DNA with levels of unmethylated and artificially methylated DNA in addition to clinical samples were measured repeatedly in different runs. Methylation values of each sample and model DNA were measured in nine replicates.

The introduction of a methylation cut-off for dichotomization of the *SHOX2* methylation levels represents a technological challenge. It needs to be ensured that the accuracy and reproducibility of the assay is sufficiently high to correctly classify patient samples which show a methylation level close to the cut-off. Accordingly, the analytical performance of the assay close to the cut-off was validated. Four PE samples from the clinical performance evaluation study as described above were selected which showed a methylation level close to the cut-off. Two samples showed a methylation level above the cut-off and two below. In addition, defined mixtures of artificially methylated DNA and unmethylated DNA were analyzed. The clinical samples and the DNA mixtures were repeatedly measured in nine repeated measurements. Each measurement was comprised of three single replicates resulting in a total of 27 replicates per samples. The measurements were conducted in three independent runs in order to determine the variability and robustness of the assay. The assay allowed for an accurate discrimination of samples showing a *SHOX2* methylation level above and below the cut-off **(** **Figure 4** **,** **Table 4**). Overall variability of *SHOX2* and *SEPT9* DNA methylation quantification was low. No significant inter-run variability was found, as all ANOVA p-values were greater than 0.05.

**Table 4:** Validation of cut-off (*SHOX2*).

	Clinical pleura samples				Model DNA with different methylation levels				
	A	B	C	D	30%	15%	7.5%	3.75%	1.875%
***SHOX2***									
Mean methylation [%]	3.55	15.54	22.99	3.45	43.63	20.84	11.44	5.59	3.27
SD	0.43	0.61	1.29	0.36	3.03	1.36	0.78	0.39	0.38
%CV	12.14	3.92	5.62	10.48	6.95	6.54	6.78	6.89	11.53
Inter-run (p-value)	0.44	0.20	0.27	0.16	0.06	0.09	0.19	0.41	0.76

Statistical analysis of repeated measurements of pleural effusion samples and model DNA samples with different methylation levels close to the cut-off.

## Discussion

In this study, DNA methylation of the *SHOX2* and *SEPT9* gene loci were shown to be highly specific biomarkers for the diagnosis of malignant pleural effusions. While the specificity was 100% for both biomarkers, the apparent overall positivity of 26% for *SHOX2* and *SEPT9* represented an improvement as compared to cytology alone. However, due to lack of a highly accurate diagnostic gold standard for the differential diagnosis of PEs, this positivity does not represent the sensitivity of the test. The true number of MPEs is most likely higher than the observed positivity since not all PEs from cancer patients are in fact MPEs. Cytology for example does not allow the differentiation between false negative MPEs and truly negative PPEs. Therefore, a positivity of 100% cannot be expected even from a diagnostic test with 100% sensitivity since its performance is always related to cytology as a gold standard. In this case the positivity can only reach the level of the portion of MPEs in the group of cancer patients since this group is comprised of patients with MPEs and PPEs. The true number of MPEs in cancer patients analyzed in this study is unclear and therefore the exact sensitivity is speculative. However, since the group of cases and the group of controls analyzed in this case control study do not differ significantly regarding the presence of benign diseases, i.e. heart failure and pneumonia, it is likely that a significant number of PEs from cancer patients were in fact paramalignant. After cancer (27%), heart failure (20%) and pneumonia (18%) are the second and third leading cause, respectively, for the development of PEs [2]. In the presented study, heart failure and pneumonia together were present in 19% of the cancer patients. Therefore, it can be concluded that a significant number of PEs in cancer patients included in this study were in fact paramalignant and caused by benign diseases. Accordingly, the comparison of the calculated sensitivity of cytological analysis with reported cytological sensitivities is not trivial. Other studies classify PEs from cancer patients with negative results in cytology already as BPE if a benign disease was diagnosed which may have caused the PE [19], [39]. However, it is not evident if this benign disease in fact caused the PE or if the PE was due to a malignancy. Therefore, the reported sensitivity of cytology in these studies is likely to be overestimated.

Furthermore, this study showed that patients with *SHOX2* or *SEPT9* positive PEs have an adverse overall survival and the respective methylation levels significantly correlate with a worse outcome. These findings indicate that *SHOX2* or *SEPT9* in fact allowed for the discrimination between PPEs and MPEs. Patients with MPEs are expected to have a worse outcome as compared to patients with PPEs due to the more advanced stage of the disease in patients with MPEs. *SHOX2* DNA methylation has been reported to detect lung cancer in bronchial aspirates with a sensitivity of 68–78% and a specificity of 95–96% [26], [27]. These studies were based on the analysis of the cellular fraction of bronchial lavage and a positive *SHOX2* methylation signal was indicative for the presence of malignant cells. From the technological point of view, the sample material was similar to the sample material from PEs and a similar sensitivity might be expected when analyzing PEs.

Besides the unavailability of a gold standard to discriminate between MPE and PPE and therefore to allow for the calculation of the sensitivity, the design of the presented study implied two major limitations. Firstly, the relatively low number of included patients limits the significance regarding sensitivity and specificity of the test. This is particularly important when regarding the determination of the specificity. A high specificity of ideally 100% is needed for each single biomarker in order to allow for the combination of several biomarkers without a loss of overall specificity. It is likely that the specificity will decrease when analyzing a higher number of control patients, since the normal population variation might be higher than observed here. This might necessitate shifting the cut-off for dichotomization of the *SHOX2* signal towards higher methylation, therefore leading to a loss of sensitivity. Methylation of *SEPT9* has been found in plasma of a significant number of patients without malignant disease [33]. Therefore, it is likely that control patients with positive *SEPT9* signal might be found when analyzing higher numbers of control patients. This would necessitate the implementation of a cut-off for *SEPT9* as well, thus potentially reducing the sensitivity. However, the presented and previous studies showed [26], [27] that the level of *SHOX2* and *SEPT9* methylation directly correlated with the likelihood of the presence of malignant cells. Therefore, the careful evaluation of the measured methylation level prior to result dichotomization might allow the clinicians to draw personalized diagnostic conclusions. The second limitation of this study is the lack of a reference opinion for the cytopathological assessment of all patient samples. It is likely that the overall sensitivity of cytology increases with the number of reference cytopathologists assessing the specimens. This could reduce the potential additive value of a biomarker test in adjunct to cytology. However, it should be considered that a clinically useful diagnostic test needs to work in standard clinical routine where the opinion of several experienced cytopathologists can hardly be obtained. DNA methylation based biomarker tests have been shown to be highly robust and reproducible [27] and therefore can smoothly be implemented into clinical routine without the need of highly experienced personnel.

34 out of 114 (30%) patient samples gave invalid results during methylation analysis due to the lack of a sufficient amount of DNA in the sample. A sufficient number of DNA copies in the sample are required in order to distinguish between false and true negative results. The results might be false negative if the cell number equivalent related to the DNA amount is too low and therefore the likelihood of the presence of DNA from malignant cells is limited as well. However, the presented study was conducted using left-over sample material which was obtained after completion of cytopathological routine diagnostics. A sampling procedure which is optimized with regard to the application to a molecular biological test will increase the number of valid results due to the availability of higher cell numbers. A negative or invalid test result does not have an impact on the diagnosis since cytology is still the gold standard and is included in the test. Accordingly, invalid and negative samples would be handled like patients samples with only cytological results available. However, because of the high positive predictive value of the assay, a positive assay result should be considered for further treatment of patients. The high number of invalid results on the other hand led to a decrease of sensitivity of the test since invalid results of cancer patient samples were treated as false negative results. Therefore, an optimized sample preparation is likely to lead to an increase of sensitivity.

Although *SHOX2* is a validated biomarker in lung cancer [26], [27], its positivity was low in pleural fluid of lung cancer patients (13%). This fact might be attributed to low DNA amounts, paramalignant effusions or the high background methylation of *SHOX2* in PEs which necessitated the definition of a high cut-off that was higher than in previous studies where plasma or bronchial aspirates were analyzed [26], [27], [28]. Further studies including larger patient cohorts are needed to address this question.

The clinical performance of *SHOX2* and *SEPT9* was similar as compared to published studies. However, the sensitivities of the tumor markers CEA, CA 125, CA 15-3, CYFRA 21-1 and mesothelin strongly depend on the tumor entity. The overall sensitivity of each marker analyzed in all cancer entities did not exceed 35% when considering 100% specificity [19]. The introduction of cut-offs and the combination of CEA, CA 125, CA 15-3, CYFRA 21-1 lead to an overall sensitivity of 54% at 100% specificity. However, the combination of *SHOX2* and *SEPT9* methylation biomarkers with additional biomarkers should be taken into consideration. Since *SHOX2* and *SEPT9* seem to be suitable biomarkers in different tumor entities a benefit regarding the overall sensitivity is possible. For example, *SEPT9* allowed for the detection of Non-Hodgkin lymphoma in one of three cases. This entity was not detected by the combination of CEA, CA 125, CA 15-3 and CYFRA 21-1 [19].

A comparison of *SHOX2* and *SEPT9* and the biomarkers NSA, TSA and CA 19-9 is difficult since the latter were only tested on primary malignancies of the bronchus or the pleura [17]. The diagnostic value of soluble mesothelin-related peptide (SMRP) was mainly investigated for the discrimination between mesothelioma and benign effusions [40], [41]. Due to its low diagnostic accuracy the use of survivin is not recommended for the discrimination between benign and malignant pleural effusions, but it may be used as a predictive or prognostic biomarker [42].

In summary, this study describes *SHOX2* and *SEPT9* as promising biomarkers for the diagnosis of MPEs and might be used in adjunct to cytological assessment. Furthermore, the methylation biomarkers *SHOX2* and *SEPT9* might improve the prognostic accuracy. In particular DNA methylation biomarkers are promising candidates for clinical application where a sensitive detection is required. Hypermethylation of *SHOX2* has previously been shown to be frequently accompanied by gene amplification leading to apparent methylation levels above 100% [25]. The high sensitivity of the *SHOX2* DNA methylation biomarker in plasma and bronchial aspirates as reported earlier [26], [27], [28] could be due to a correlation of DNA methylation and locus amplification in malignant cells.

The assay described here may be used as a diagnostic adjunct to existing clinical and cytopathological investigations in patients with a PE and might allow for an improved patient management.

## Supporting Information

Figure S1 **Analytical assay performance.** Analytical performance of qPCR assay for accurate and sensitive detection of *SHOX2* and *SEPT9* DNA methylation. Shown are means of triplicate measurements.
(TIF)

Table S1 **Oligonucleotide specifications.** Sequences, labels and final concentrations of the oligonucleotides used in the quantitative real-time PCR for measuring DNA methylation of *SHOX2* and *SEPT9* in PEs. Genomic localizations of amplicons refer to assembly GRCh37/hg19.
(DOC)

Table S2 **Tumor site specific clinical performance.** Positivity of the developed assay in PEs from 58 cancer patients with respect to the primary tumor.
(DOC)
