# Supplementary figures and images for: Diagnostic and Prognostic Value of SHOX2 and SEPT9 DNA Methylation and Cytology in Benign, Paramalignant and Malignant Pleural Effusions
Source: PLoS One. 2013 Dec 27;8(12):e84225. doi: 10.1371/journal.pone.0084225 (PMC3874014; doi:10.1371/journal.pone.0084225)

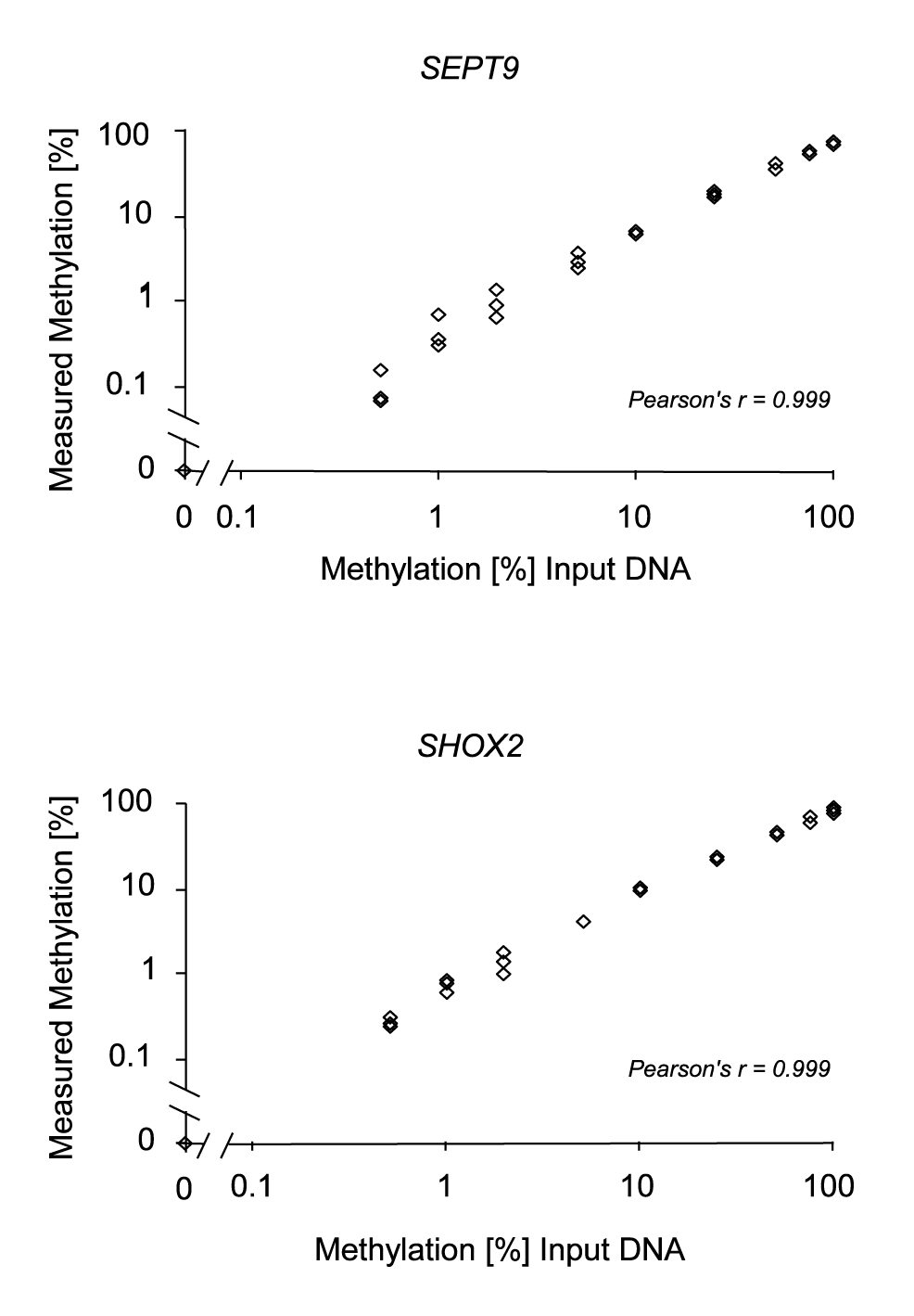

Supplement: Figure S1 — Analytical assay performance. Analytical performance of qPCR assay for accurate and sensitive detection of SHOX2 and SEPT9 DNA methylation. Shown are means of triplicate measurements. (TIF) [file pone.0084225.s001.tif]
