# Supplementary material for: Diagnostic and Prognostic Value of SHOX2 and SEPT9 DNA Methylation and Cytology in Benign, Paramalignant and Malignant Pleural Effusions
Source: PLoS One. 2013 Dec 27;8(12):e84225. doi: 10.1371/journal.pone.0084225 (PMC3874014; doi:10.1371/journal.pone.0084225)
Supplement: Table S1 — Oligonucleotide specifications. Sequences, labels and final concentrations of the oligonucleotides used in the quantitative real-time PCR for measuring DNA methylation of SHOX2 and SEPT9 in PEs. Genomic localizations of amplicons refer to assembly GRCh37/hg19. (DOC) [file pone.0084225.s002.doc]

| **Oligonucleotide** | **Concentration in PCR [µM]** | **Sequence 5’ 🡪 3’** |
| --- | --- | --- |
| ***SHOX2*** |  |  |
| chr3:157821339-157821449 |  |  |
| Forward Primer | 0.4 | GTTTTTTGGATAGTTAGGTAAT |
| Forward Blocker | 0.75 | TAATTTTTGTTTTGTTTGTTTGATTGGGGTTGTATGA-SpacerC3 |
| Reverse Primer (MSP-Primer) | 0.4 | TAACCCGACTTAAACGACGA |
| Hydrolysis Probe | 0.3 | 6-FAM-CTCGTACGACCCCGATCG-BBQ-650 |
|  |  |  |
| ***SEPT9*** |  |  |
| chr17:75369563-75369622 |  |  |
| Forward Primer | 0.4 | AAATAATCCCATCCAACTA |
| Reverse Primer | 0.4 | GTTGTTTATTAGTTATTATGT |
| Reverse Blocker | 0.75 | GTTATTATGTTGGATTTTGTGGTTAATGTGTAG-Spacer C3 |
| Hydrolysis Probe | 0.3 | JOE-TTAACCGCGAAATCCGAC-BHQ1 |
|  |  |  |
| ***ACTB*** |  |  |
| chr7:5571731-5571859 |  |  |
| Forward Primer | 0.225 | GTGATGGAGGAGGTTTAGTAAGTT |
| Reverse Primer | 0.225 | CCAATAAAACCTACTCCTCCCTTAA |
| Hydrolysis Probe | 0.3 | Cy5-ACCACCACCCAACACACAATAACAAACACA-BBQ650 |
