# Supplementary material for: Diagnostic and Prognostic Value of SHOX2 and SEPT9 DNA Methylation and Cytology in Benign, Paramalignant and Malignant Pleural Effusions
Source: PLoS One. 2013 Dec 27;8(12):e84225. doi: 10.1371/journal.pone.0084225 (PMC3874014; doi:10.1371/journal.pone.0084225)
Supplement: Table S2 — Tumor site specific clinical performance. Positivity of the developed assay in PEs from 58 cancer patients with respect to the primary tumor. (DOC) [file pone.0084225.s003.doc]

|  | **Diagnostic Result (Positive PEs from Cancer Patients)** | | | | |
| --- | --- | --- | --- | --- | --- |
| **Primary Tumor** | ***SHOX2*** | ***SEPT9*** | ***SEPT9* or *SHOX2*** | **Cytology** | ***SEPT9* or *SHOX2* or Cytology** |
| **Digestive System** | 4/17 (24%) | 6/17 (35%) | 7/17 (41%) | 4/17 (24%) | 7/17 (41%) |
| Stomach | 2/3 (33%) | 2/3 (33%) | 2/3 (33%) | 2/3 (33%) | 2/3 (33%) |
| Small Intestine | 1/1 (100%) | 0/1 (0%) | 1/1 (100%) | 0/1 (0%) | 1/1 (100%) |
| Colon | 1/4 (25%) | 1/4 (25%) | 1/4 (25%) | 0/4 (0%) | 1/4 (25%) |
| Anus, Anal Canal, & Anorectum | 0/1 (0%) | 0/1 (0%) | 0/1 (0%) | 0/1 (0%) | 0/1 (0%) |
| Liver & Intrahepatic Bile Duct | 0/7* (0%) | 2/7* (29%) | 2/7* (29%) | 1/7* (14%) | 2/7* (29%) |
| Pancreas | 0/1 (0%) | 1/1 (100%) | 1/1 (100%) | 1/1 (100%) | 1/1 (100%) |
|  |  |  |  |  |  |
| **Respiratory System** ¶ | 1/10 (10%) | 3/10 (30%) | 3/10 (30%) | 1/10 (10%) | 3/10 (30%) |
| Larynx | 0/2 (0%) | 0/2 (0%) | 0/2 (0%) | 0/2 (0%) | 0/2 (0%) |
| Lung & Bronchus | 1/8* (13%) | 3/8* (38%) | 3/8* (38%) | 1/8* (13%) | 3/8* (38%) |
|  |  |  |  |  |  |
| **Bones & Joints** | 0/1* (0%) | 0/1* (0%) | 0/1* (0%) | 0/1* (0%) | 0/1* (0%) |
|  |  |  |  |  |  |
| **Skin (Excluding Basal & Squamous)** | 0/1 (0%) | 0/1 (0%) | 0/1 (0%) | 0/1 (0%) | 0/1 (0%) |
| Melanoma-skin | 0/1 (0%) | 0/1 (0%) | 0/1 (0%) | 0/1 (0%) | 0/1 (0%) |
|  |  |  |  |  |  |
| **Breast** | 2/11* (18%) | 1/11* (9%) | 3/11* (27%) | 5/11* (46%) | 6/11* (55%) |
|  |  |  |  |  |  |
| **Genital System** | 0/7 (0%) | 0/7 (0%) | 0/7 (0%) | 2/7 (29%) | 2/7 (29%) |
| Uterine Cervix | 0/1* (0%) | 0/1* (0%) | 0/1* (0%) | 0/1* (0%) | 0/1* (0%) |
| Ovary | 0/5 (0%) | 0/5 (0%) | 0/5 (0%) | 2/5 (40%) | 2/5 (40%) |
| Prostate | 0/1 (0%) | 0/1 (0%) | 0/1 (0%) | 0/1 (0%) | 0/1 (0%) |
|  |  |  |  |  |  |
| **Urinary System** | 0/5 (0%) | 1/5 (20%) | 1/5 (20%) | 0/5 (0%) | 1/5 (20%) |
| Kidney & Renal Pelvis | 0/4 (0%) | 1/4 (25%) | 1/4 (25%) | 0/4 (0%) | 1/4 (25%) |
| Ureter & other Urinary Organs | 0/1 (0%) | 0/1 (0%) | 0/1 (0%) | 0/1 (0%) | 0/1 (0%) |
|  |  |  |  |  |  |
| **Brain & other Nervous System** | 0/1 (0%) | 0/1 (0%) | 0/1 (0%) | 0/1 (0%) | 0/1 (0%) |
|  |  |  |  |  |  |
| **Endocrine System** | 0/2 (0%) | 0/2 (0%) | 0/2 (0%) | 1/2 (50%) | 1/2 (50%) |
| Thyroid | 0/2 (0%) | 0/2 (0%) | 0/2 (0%) | 1/2 (50%) | 1/2 (50%) |
|  |  |  |  |  |  |
| **Lymphoma** | 0/5 (0%) | 1/5 (20%) | 1/5 (20%) | 0/5 (0%) | 1/5 (20%) |
| Non-Hodgkin Lymphoma | 0/3* (0%) | 1/3* (33%) | 1/3* (33%) | 0/3* (0%) | 1/3* (33.3%) |
| Myeloma | 0/2 (0%) | 0/2 (0%) | 0/2 (0%) | 0/2 (0%) | 0/2 (0%) |
|  |  |  |  |  |  |
| **Leukemia** | 0/1 (0%) | 0/1 (0%) | 0/1 (0%) | 0/1 (0%) | 0/1 (0%) |
| Acute Myeloid Leukemia | 0/1* (0%) | 0/1* (0%) | 0/1* (0%) | 0/1* (0%) | 0/1* (0%) |
| Chronic Myeloid Leukemia | 0/1* (0%) | 0/1* (0%) | 0/1* (0%) | 0/1* (0%) | 0/1* (0%) |
|  |  |  |  |  |  |
| **Other & Unspecified Primary Sites** | 0/1 (0%) | 1/1 (100%) | 1/1 (100%) | 0/1 (0%) | 1/1 (100%) |

*one patient with lung, uterine cervix cancer and non-Hodgkin lymphoma, one patient suffering from lung and liver cancer, one patient with breast and bone cancer, and one patient with acute and chronic myeloid leukemia and breast cancer.
